# Leveraging cryoablation and checkpoint inhibitors for high-risk triple negative breast cancer

**DOI:** 10.3389/fimmu.2023.1258873

**Published:** 2023-10-04

**Authors:** Flávia Sardela de Miranda, Maribel Castro, Nicole Remmert, Sharda P. Singh, Rakhshanda Layeequr Rahman, Michael W. Melkus

**Affiliations:** ^1^Department of Surgery, School of Medicine, Texas Tech University Health Sciences Center, Lubbock, TX, United States; ^2^Department of Immunology and Molecular Microbiology, School of Medicine, Texas Tech University Health Sciences Center, Lubbock, TX, United States; ^3^Breast Center of Excellence, Texas Tech University Health Sciences Center, Lubbock, TX, United States; ^4^Department of Internal Medicine, School of Medicine, Texas Tech University Health Sciences Center, Lubbock, TX, United States

**Keywords:** breast cancer, cryoablation, abscopal effect, immune checkpoint inhibitors, immune response, drug delivery

## Abstract

Breast cancer is the second most common cancer among women in the United States in which the standard of care treatment is surgery with adjunctive therapy. Cryoablation, which destroys the tumor using extremely cold temperatures while preserving the potential tumor antigens, is a promising alternative to surgical resection. It is less invasive, cosmetically appeasing, cost-effective, and capable of contributing to the abscopal effect – the immune response targeting potential distant metastasis. However, to maximize the immunologic benefit of cryoablation in biologically high-risk breast cancers, combination with therapies that enhance immune activation, such as immune checkpoint inhibitors (ICIs) may be necessary. This mini review describes the fundamentals of cryoablation and treatment with ICIs, as well as discuss the caveats in both strategies and current clinical trials aimed to improve this approach to benefit patients.

## Introduction

The current standard of breast cancer therapy involves local treatment via surgical resection (lumpectomy or mastectomy) of the tumor and regional lymph nodes. Following surgery, patients may undergo adjunctive systemic treatment with chemotherapy, immunotherapy, and/or endocrine therapy. Despite recent advances in therapeutic options, select subtypes of breast cancers, such as triple-negative breast cancers (TNBC) remain at high risk for early metastasis and disease recurrence (1). TNBCs by virtue of being estrogen receptor (ER), progesterone receptor (PR), and human epidermal growth factor receptor 2 (HER2) negative, lack known therapeutic targets. Therefore, it is essential to develop innovative strategies for the treatment of TNBCs.

Cryoablation, the process of destroying target tissue by exposing it to freeze/thaw cycles, has become a promising alternative to surgical resection. The procedure is performed under local anesthesia and entails the insertion of a cryoprobe into the tumor with guidance from imaging techniques such as ultrasound, CT, or magnetic resonance. Next, rapid freeze/thaw/freeze cycles are performed, and an ice-ball forms engulfing the tumor along with a margin of normal tissue. These cycles expose the tumor to extremely cold temperatures (≤ -40°C), forming ice crystals, which damage tumor cell membranes, and loss of blood supply due to endothelial cell dysfunction. Additionally, during the freeze phase, the water in the extracellular space will freeze faster than it does in the intracellular compartment, setting up an osmotic gradient that will drive fluids out of the cells; during the thawing phase, the rapid reversal of the gradient results in water quickly entering the cells, contributing to cell rupture. Together, these mechanisms lead to the destruction and necrosis of the tumor (2).

**Figure 1:**
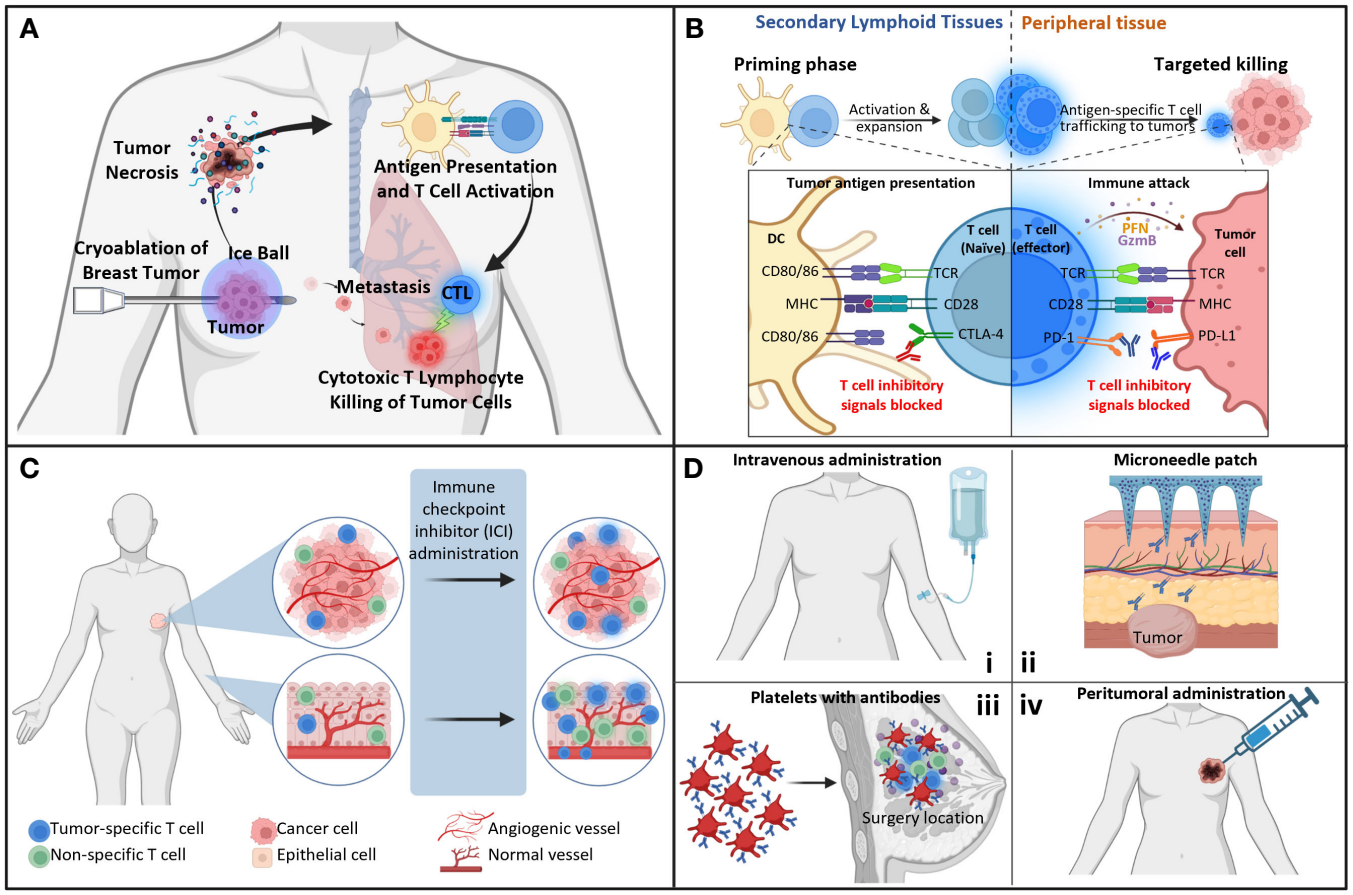
Immunological aspects of immunotherapies. **(A)** The abscopal effect. Cryoablation of primary tumor induces an antitumor immune response that can target distant metastasis. **(B)** Checkpoint inhibitors. Immune checkpoint inhibitors act at different T cell stages. Anti-CTLA-4 acts mainly in the lymph nodes and spleen, where it inhibits the binding of CTLA-4 to B7, blocking the inhibitory signal that would prevent the T cell from being activated. Meanwhile, anti-PD-1 and anti-PD-L1 act mainly in the tumor, where they prevent the binding of one molecule to the other, blocking the inhibitory signal that would cause T cells to be exhausted. **(C)** Immune-related adverse effects. Studies have shown that after immune checkpoint inhibitor administration to patients, the population of anti-tumor specific T cells is enriched in the tumor, as well as non-tumor specific but self-antigen specific T cells in tissues that may lead to auto-immune reactions post-treatment. These cells could have originated from the quiescent resident cells in the organ, or they could have migrated from the tumor to the affected tissue. **(D)** Checkpoint inhibitor delivery methods. (i) Systemic administration of low/ultralow doses, (ii) . microneedle patch to the skin, (iii). intravenous administration of platelets conjugated to the antibodies, (iv). direct injection into tumor. CTL, cytotoxic; DC, dendritic cell; MHC, major histocompatibility complex. Created with BioRender.com.

In addition to the mechanical damage to the tumor cells, cryoablation can potentially generate a systemic tumor-specific immune response, known as the abscopal effect – defined as the phenomenon where local treatment of primary tumor leads to an increased systemic immune response, thereby affecting potential metastases (**Figure 1A**). It is hypothesized that the release of tumor-associated antigens by cryoablation, cellular stress signals, and inflammatory cytokines facilitate this process, in opposition to traditional resection, where the tumor is completely removed and tumor antigens are not preserved (3). The first reported use of cryoablation on breast cancer was in 1985 on a woman with a 1-2 cm palpable tumor (4). The procedure was successful, and the patient maintained disease-free at a 2-year follow-up. Recent advances in immune therapies for various cancers have led to enhanced interest in investigating the role of cryoablation in inducing systemic immune responses and exploring its use in clinical practice. Experiments by Sabel (5), Khan (6) and Wu (7) explored the immune response to cryoablation and the abscopal effect in mice models of cancer. Sabel et al. reported in a breast cancer model the induction of tumor-specific T-cell response in tumor-draining lymph nodes and increased systemic natural killer cell activity post cryoablation (5). Khan et al. concluded that cryoablation induced a tumor-specific tumor-infiltrating lymphocytes (TIL) response (6). Wu et al. noted in a mouse colon cancer model, the induction of immune effector cells, a decrease in immunosuppressive cells and an overall enhancement of anti-tumor immunity in distant tumor microenvironment post cryoablation (7).

In clinical studies, the use of cryoablation to treat fibroadenomas has shown evidence to effectively reduce tumor volume, produce less pain, and obtain good cosmesis (8). Further studies have been done to assess cryoablation’s effectiveness in early-stage breast cancers. The American College of Surgeons Oncology Group (ACOSOG) conducted a phase II clinical trial where eighty-six patients with unifocal invasive ductal carcinoma < 2 cm, less than 25% intraductal component, and tumor enhancement on MRI underwent cryoablation with subsequent surgical resection within 28 days. Complete ablation was defined as no remaining invasive breast carcinoma or ductal carcinoma *in situ* on pathological examination of the targeted lesion. The study demonstrated successful ablation of 75.9% of the cancers, with 100% successful ablation in all tumors smaller than 1 cm (9) (**Table 1**).

**Table 1:** Breast Cancer Cryoablation Clinical Trials.

Trial Name/#	Title	Goal/Purpose	Tumor Type	Enrollment	Interventions	Primary outcome measure	Trial dates
Cryoablation alone							
ACOSOG Alliance Z1072/ NCT00723294	Cryoablation Therapy in Treating Patients with Invasive Ductal Breast Cancer	Determine the rate of complete tumor ablation in patients treated with cryoablation	• Unifocal primary invasive ductal breast carcinoma • Max size ≤ 2.0 cm	• ≥ 18 years old • 99 participants	• Cryoablation • Surgical resection	• Rate of complete tumor ablation [Time Frame: Up to 14 days post- surgery] • No remaining invasive or *in situ* carcinoma upon pathological examination	Sept 2008 - Sept 2013
ICE3- Trial/ NCT02200705	Cryoablation of Low-Risk Small Breast Cancer- Ice3 Trial	Evaluate efficacy of cryoablation without lumpectomy and its impact on local and distant recurrence	• Early-stage breast cancers • HR+ PR+, HER2- • Max size ≤ 1.5 cm	• ≥ 50 years old • 208 participants	• Cryoablation	• Local Inbreast Breast Tumor Recurrence (IBTR) rate at 6 months	Oct 2014 – Dec 2024
COOL-IT/NCT05505643	Cryoablation vs Lumpectomy in T1 Breast Cancers	Comparing cryoablation to lumpectomy for better disease control, complications, and quality of life	• Invasive ductal carcinoma Luminal Type A. • HR+ PR+, HER2-	• ≥ 50 years old • 256 participants	• Cryoablation • Endocrine therapy *Versus* • Lumpectomy	• Safety lead-in accessed through 30 days • Randomized trial: cryo vs resection recurrence at 5 years	June 2023 –Dec 2030
Cryoablation in combination with checkpoint inhibitor(s)							
NCT01502592	Pre-Operative, Single-Dose Ipilimumab and/or Cryoablation in Early Stage/Resectable Breast Cancer	Pilot study evaluating the safety and tolerability of pre-operative, single dose ipilimumab and/or cryoablation	• Invasive • Adenocarcinoma • Max size ≤ 1.5 cm	• ≥ 18 years old • 19 participants	• Ipilimumab • Cryoablation	Safety will be evaluated for all treated subjects using National Cancer Institute (NCI) Common Terminology Criteria for Adverse Events v4.0 (CTCAE).	Dec 2011 - Dec 2014
NCT02833233	A Study of Pre-Operative Treatment with Cryoablation and Immune Therapy in Early-Stage Breast Cancer	Evaluate the safety of combining “cryoablation” and “immune therapy” in women with curable early-stage breast cancer	• Invasive • Adenocarcinoma • Max size ≤ 1.5 cm	• ≥ 18 years old • 5 participants	• Ipilimumab • Nivolumab • Cryoablation • Surgical resection	Safety will be evaluated for all treated subjects using National Cancer Institute (NCI) Common Terminology Criteria for Adverse Events v4.0 (CTCAE).	June 2016 - June 2023
NCT03546686	Peri-Operative Ipilimumab + Nivolumab and Cryoablation in Women with Triple-Negative Breast Cancer	Determine impact of pre-operative cryoablation, ipilimumab and nivolumab on 3-year Event Free Survival (EFS), in women with triple negative breast cancer after taxane-based neoadjuvant chemotherapy	• Invasive adenocarcinoma • HR- PR-, HER2- (triple-negative) • Size ≥ 1.0 cm	• ≥ 18 years old • 80 participants	• Ipilimumab • Nivolumab • Cryoablation • Standard of care definitive surgery	Event-Free Survival [Time Frame: 36 Months]	June 2019 - June 2026
LOGIC/ NCT05806385	Grouping Immune modulation With Cryoablation (LOGIC) for Breast Cancers (LOGIC)	Determine if cryoablation with pembrolizumab for local control in high-risk triple negative breast cancer is superior to surgical resection alone or cryoablation alone in generating antitumor immune response.	• Invasive carcinoma • ER -, PR-, HER2- (triple negative) • Unifocal disease visible on ultrasound	• ≥ 18 years old • 36 participants	• Pembrolizumab • Cryoablation • Standard of care definitive surgery * Versus* • Resection alone • Cryoablation alone	• Rate of Complete Pathological Response • Tumor I nfiltrating Lymphocytes Scores	Jan 2024 - June 2027

Complete list of registered breast cancer cryoablation clinical trials: https://clinicaltrials.gov/.

HR, hormone receptor.

ACOSOG Z1072 was followed by the non-resection trial called “Cryoablation of Low-Risk Small Breast Cancer (ICE3)”, evaluating the recurrence rate and efficacy of cryoablation without lumpectomy in 208 woman women with low-risk breast cancer (hormone receptor positive, HER2-) (10). The ICE3 trial released a 3-year interim analysis showing an ipsilateral breast tumor recurrence of 2.06% (2/194 patients) at a mean follow-up of 34.83 months (11). Cryoablation vs Lumpectomy in T1 Breast Cancers (COOL-IT) trial will assess the safety, disease control, complication rates and quality of life in patients with low risk breast cancers treated with cryoablation or lumpectomy (12). The design and plan of these clinical studies on cryoablation in breast cancer are depicted in **Table 1**. The final outcomes of these trials will help further guide the future of cryoablation in treating low-risk breast cancer.

## Pros and cons of breast cancer cryoablation

Treatment of breast carcinoma has evolved in strides since the 1800s when radical mastectomies were the gold standard. Since then, breast cancer treatment has been geared toward preserving tissue and overall cosmesis. Cryoablation is an optimal option for both a minimally invasive procedure with proven efficacy, and it has several advantages over surgery (10, 11). Compared to surgical resection, cryoablation can be done in an outpatient setting, with the procedure usually lasting about 30 minutes to an hour, and it only requires a 3mm incision to insert the cryoprobe percutaneously under local anesthesia, which is well tolerated by patients. The absence of a large incision and general anesthesia lowers the risk of infection and complications of surgical resection, as well as optimizing time efficiency, producing less pain, and resulting in good cosmesis (8). Additionally, cryoablation provides an alternative treatment for older patients who are poor surgical candidates or are reluctant to undergo surgery. Lastly, compared to lumpectomy, cryoablation treatment is a cost-effective option for patients, with our study showing lower cost of care by 86.85% and higher overall patient satisfaction (13).

Disadvantages of cryoablation include the learning curve for developing expertise in performance of the procedure and interpretation of ultrasonographic imaging. Other imaging techniques for cryoablative targets have been reported including CT scans and MRIs (14, 15); however, these are more cumbersome and expensive strategies. Additionally, cryoablation is not without possible adverse effects. Although rare, adverse effects include skin necrosis, fat necrosis, pectoralis muscle necrosis, and infection (16). There is only limited clinical data on cryoablation use for high-risk breast cancers, with most ongoing trials focusing on older women with small, low-risk breast tumors. Finally, not all individuals treated with cryoablation reliably elicit the abscopal effect (17).

## Combination of cryoablation with checkpoint inhibitors

The use of immune checkpoint inhibitors (ICIs) as immunotherapy for breast cancer is a promising strategy as it increases the capacity of the immune system to recognize tumor cells and potentially prevent recurrence. In recent years, there has been a growing body of research on the efficacy of checkpoint inhibitors with breast cancers, specifically antibodies against cytotoxic T-lymphocyte antigen-4 (CTLA-4), programmed cell death-1 protein (PD-1) and programmed cell death ligand-1 (PD-L1) (**Figure 1B**). A meta-analysis by Qi et al. investigated the efficacy and safety of anti-PD-1/PD-1 monotherapy for metastatic breast cancer (18). Overall, global analysis demonstrated that complete response was 1.26%, partial response was 7.65% and objective response rate was 9.85%. Efficacy between PD-L1 positive and PD-L1 negative groups was also compared, and they found that PD-L1-positive groups had an objective response rate of 10.62% compared to 3.07% in the PD-L1-negative group. Most recently in July 2021, the Food and Drug Administration approved Keytruda (pembrolizumab), an anti-PD-1 antibody, as adjuvant treatment for high-risk, early-stage TNBC, based on the results from KEYNOTE-522 (19). There have been a greater number of clinical trials investigating anti-PD-1/PD-L1 than anti-CTLA-4 agents. Anti-CTLA-4 antibody was the first ICI to be developed, however, clinical trials of this antibody against high-grade breast cancer are limited. A phase 1 study with 26 patients with advanced hormone-responsive breast cancer investigated the effects of tremelimumab in combination with exemestane. This study showed that there were no partial or complete objective responses, but disease stability after treatment was seen in 42% of patients (20).

The limited efficacy of monotherapy with ICIs against high-risk breast cancers is likely due to the low mutational burden of tumors, hostile microenvironment, and lack of antigen-specific T-cells (21, 22). The ability of cryoablation to release tumor-associated antigens and create a robust immune response has led many studies to hypothesize that its combination with ICIs could result in a synergistic effect against tumor cells. A pilot study of preoperative ipilimumab (anti-CTLA-4) and cryoablation in 19 women with early-stage breast cancer was conducted to determine the safety and tolerability of monotherapy and combinational therapy (23) (**Table 1**). This study showed that the combination of ipilimumab and cryoablation was safe and tolerated by participants. Combinational therapy also demonstrated favorable systemic immunological effects, including sustained peripheral elevations in Th1-type cytokines and an elevation in activated and proliferative CD4+ and CD8+ T cells. Additionally, intratumoral analysis of combinational therapy showed greater proliferative CD4+ and CD8+ T cells. Though this study showed favorable peripheral and intratumoral immune activation, it is limited by its sample size as well as its limited scope of cancer subtypes. Currently, there are three clinical trials assessing cryoablation in combination with ICIs in breast cancer (**Table 1**): A Study of Pre-Operative Treatment with Cryoablation and Immune Therapy in Early Stage Breast Cancer (24), Peri-Operative Ipilimumab + Nivolumab and Cryoablation in Women with Triple-negative Breast Cancer (25), and Grouping Immune-modulation With Cryoablation (LOGIC) for Breast Cancers (LOGIC) (26). The first two studies are using the combination of cryoablation with ipilimumab (anti-CTLA-4) and nivolumab (anti-PD-1); the first study’s goal is to measure adverse effects, while the second study’s objective is to measure event-free survival. Meanwhile, the third study mentioned above (LOGIC) is studying the combination of cryoablation with prembolizumab (anti-PD-1) for high-risk triple negative breast cancer, with the goal to measure the rate of complete pathologic response and the impact on infiltration of tumors by lymphocytes.

## Side effects of checkpoint inhibitors

The use of checkpoint inhibitors has shown benefits for the anti-tumor response in various cancers but has also resulted in toxicity that differed from the side effects of conventional anticancer treatments such as cytotoxic chemotherapy; treatment with anti-CTLA-4 or anti-PD-1 antibodies are associated with inflammatory side effects, named together as immune-related adverse effects (irAEs) (27). These irAEs are associated with the role of the adaptive immune system, which involves the activation of T and B lymphocytes. During development, the T and B cells that have receptors that recognize self-peptides are tolerized against them to avoid the development of an immune reaction against self-tissues (avoiding autoimmunity), in a process that involves passive and active suppression of the immune system. The establishment and maintenance of this homeostasis are mediated by, among other factors, CTLA-4 and PD-1. The use of checkpoint inhibitors against these molecules can improve the antitumor response as discussed above by enhancing the activity of effector cells, however, since the blockade of these regulatory pathways is not antigen-specific, it can also result in autoimmunity by releasing the brakes previously established for auto-peptides (28). Indeed, both anti-CTLA-4 and anti-PD-1 have been associated with irAEs in different studies (29–35).

In melanoma patients, the toxicity of ipilimumab was shown to be dose-dependent (30, 31), while the high-grade toxicity observed with anti-PD-1/PD-L1 therapy did not seem to be dose-dependent and occurred at lower percentages than in the anti-CTLA-4 treatment (33–35). The differences in the frequency of irAEs could be explained by the different mechanisms of action between the blockers; while CTLA-4 blockade leads to nonspecific expansion of existing T cell clones and suppression of regulatory T cells (35), PD-1 signaling inhibition stimulates clonal expansion of T cell clones present in the tumor site (36), which intuitively could mean that there’s less chance of having auto-reactive activated T cells.

Management of irAEs can be done by the administration of anti-inflammatory medication, such as steroids, similar to what is done for auto-immune diseases. Yet, there is concern about whether the use of anti-inflammatory medication to treat irAEs could also lead to decreases in the effectiveness of immunotherapy since studies with different types of cancer - including melanoma, renal, gastric, non-small cell lung cancer (NSCLC), and breast cancer - have shown that patients that better responded to anti-CTLA-4 or anti-PD-1 therapy were also the patients who developed irAEs (37–43). Therefore, uncoupling the anti-tumor immune response from the anti-patient immune activation is the main challenge immunotherapy currently faces.

While Horvat et al. (44) observed in their study no differences in overall survival and time to treatment failure when ipilimumab-treated melanoma patients were stratified by the presence or absence of irAEs of any grade and by the administration of systemic corticosteroids to treat an irAE, other studies came across different results by further stratifying patients. Faje et al. showed that, in a cohort of 98 patients with melanoma who had ipilimumab-induced hypophysitis, high doses of glucocorticoids reduced survival when compared to low doses of the medication (37). Additionally, a systematic review and meta-analysis of published studies performed by Petrelli et al. (45) concluded that patients taking steroids for any reason were at increased risk of death and progression compared to those not taking the medication; however, in the subgroup analysis, the greatest negative effect for overall survival was evident in patients taking steroids for supportive care and brain metastasis, while the effect of steroids administration to mitigate irAEs did not negatively affect overall survival.

In conjunction, these studies illustrate that to understand the impact of steroids on the effectiveness of immunotherapy, the dose and the underlying patient health need to be taken into consideration. The study by Ricciuti and collaborators in a cohort of immunotherapy-treated NSCLC patients further contributes to this philosophy. Their results showed that subjects who received ≥10 mg of prednisone at the time of immunotherapy initiation had shorter median progression-free survival (mPFS) and median overall survival (mOS) than patients who received 0 to <10 mg of prednisone. However, patients who received ≥10 mg prednisone for palliative indications had shorter mPFS and mOS than patients who received ≥10 mg prednisone for cancer-unrelated reasons and patients receiving 0 to <10 mg prednisone, who had no differences between one another (46).

The infiltration of tissues with activated T cells is a hallmark of irAEs (27). The connection between ICI treatment and irAEs is evidenced by studies showing the presence of shared T cell clones in both the tumor and the tissue affected by the auto-immunity reaction after ICI (47, 48) and studies showing that the TCR from the auto-immunity associated CD8+ T cells originates from tissue-resident populations (49) (**Figure 1C**). The most common toxicities occur at barrier sites, such as the skin, lungs, and gastrointestinal mucosa, or endocrine organs, although any other organ can be affected (27, 50). Strategies to bypass immune-related toxicity are needed, and very promising approaches are the use of low/ultralow doses of ICIs and local delivery systems. The combination of cryoablation to either strategies hold promise for the future, as it would still generate an improved abscopal effect, while causing less adverse events.

## Low/ultralow doses of ICIs

An alternative clinical approach to decrease the incidence and severity of irAEs is the use of low/ultralow doses of ICIs (**Figure 1Di**). Indeed, several clinical studies using low dose ICIs have demonstrated to be effective with lower toxicity, compared to recommended doses of ICIs (51–54). Kleef et al. first adopted the low-dose ICI rationale for stage IV cancer patients, by combining an off label low-dose of anti-CTLA-4 plus anti-PD-1 antibody blockade with hyperthermia and individualized dosing of IL-2 treatment (55). The synergism of the various T-cell stimulatory effects was demonstrated in a heavily pretreated TNBC patient, with far advanced pulmonary metastasis and severe shortness of breath, who had exhausted all conventional treatment; the patient went into complete remission of her lung metastasis and all cancer-related symptoms vanished with transient World Health Organization (WHO) Toxicity Scale grade 1 and 2 diarrhea and skin rash (55).

Moreover, an off-label low-dose ICIs protocol of ipilimumab (0.3 mg/kg) plus nivolumab (0.5 mg/kg) has already proven safe and effective in 131 unselected stage IV cancer patients with 23 different histological types of cancer (including 42 breast cancer patients) who exhausted all conventional treatments (56). During a follow-up period of up to 5 years, irAEs of WHO grade 1, 2, 3 and 4 were observed in 22.66%, 16.03%, 6.11%, and 2.29% of patients, respectively, meaning that less than half of the patients (48.09%) experienced irAEs of any grade (56).

Evidence from other cancer types also agrees with the above-mentioned findings. Results from a randomized clinical trial demonstrated a significant and clinically meaningful benefit from incorporating ultralow dose nivolumab into the treatment of patients with advanced head and neck cancer, with no increases in the development of adverse events (57). Additionally, a retrospective study of NSCLC found no significant differences in OS, PFS and high grade irAEs between patients who received 100 mg or 200 mg of pembrolizumab (51). Therefore, the combination of cryoablation with low-dose ICIs is also a promising strategy for high-risk BC that provides the benefit of being readily available for use and lowering treatment costs. Nevertheless, lowering the treatment dose of ICIs does not completely avoid the generation of irAEs, as demonstrated by Kleef et al. (56) and, therefore, the development of local delivery systems is still promising to further decrease the occurrence of adverse events.

## Local delivery of checkpoint inhibitors

The local delivery of ICIs could decrease the frequency and severity of irAEs, while also improving the efficacy of the anti-tumor response. In the past few years, scientists have explored various delivery methods for different types of cancer (**Figure 1Dii, iii, iv**). For example, Hayne et al. and Meghani et al. focused on the local delivery of ICI in bladder cancer through intravesical administration (58) or urothelial injection (59). While the results of urothelial injection are not available yet, intravesical administration presented low systemic absorption and showed to be safe, feasible and capable of eliciting strong immune responses in the small cohort recruited in this study (58). For melanoma, Wang and colleagues developed a biodegradable microneedle patch, composed of hyaluronic acid integrated with pH-sensitive dextran nanoparticles that encapsulate anti-PD-1, for controlled delivery of this ICI into the tumor; the results of the pre-clinical research showed enhanced retention of anti-PD-1 antibodies in the tumor and increased immune responses, compared to the intravenous (IV) administration of anti-PD-1 (60). These results are very promising, and further studies are needed to compare the development of irAEs in subjects receiving systemic anti-PD-1 versus controlled release with the microneedle patch.

For breast cancer specifically, the intravenous administration of platelets conjugated to PD-L1 blocking antibodies post-surgery in a murine model of this disease led to decreased recurrence and metastasis levels, and improved survival (61). The logic behind the use of platelets lies in the fact that they are known to accumulate in surgical sites and interact with circulating tumor cells; using platelets as a delivery platform would allow better interaction with specific targets, while also possibly decreasing the occurrence of irAEs since most of the platelets would accumulate near the tumor. In fact, Gurbatri and collaborators showed in the 4T1 mouse model of breast cancer that local delivery of anti-PD-L1 with the use of synchronized lysis circuit (SLC) plasmid system led to no significant toxicities, while the systemic administration of anti-PD-L1 led to severe toxicities and death (62).

Additionally, Ishihara et al. observed increased tumor retention and decreased plasma concentration of ICIs when associating them with the matrix binding protein PIGF2 (placental growth factor 2) and delivering the complex peritumorally, compared to intraperitoneal or peritumoral administration of unconjugated antibodies, besides resulting in delayed tumor growth and improved survival in a genetically engineered model of breast cancer (63). Interestingly, the treatment also led to abscopal effects. Another approach used in a different study was the injection of a reactive oxygen species (ROS)-degradable hydrogel that encapsulated gemcitabine and anti-PD-L1 antibody into the mammary fat tissue of a murine model of low-immunogenic breast cancer (4T1); the ROS-induced degradation of the hydrogel led to the release of gemcitabine, causing tumor cell death and creating an immunogenic microenvironment, which was accompanied by a delayed release of anti-PD-L1, resulting in delayed tumor growth and improved survival. Moreover, there were no obvious toxicity events observed (64). These results further support the association of cryoablation with a local delivery system for immune checkpoint inhibitors, which holds promise for the future (65).

## Summary

Cryoablation is a promising minimally invasive and safe approach for treating breast cancer associated with reduced cost, maintenance of cosmetics, and the possibility to contribute to a systemic anti-tumor response by allowing the release of intact tumor antigens for the immune system to recognize. Though already approved to treat low-risk breast tumor, the technique needs to be further advanced to achieve success in high-risk TNBC. Challenge with this subtype of cancer is the generation of a potent enough systemic response that targets potential distant metastasis and circulating cancer cells. The combination of cryoablation with ICIs can potentially address this challenge by combining the increased immunogenicity achieved by cryoablation with the improved activation of immune cells led by the inhibition of checkpoint molecules. However, the systemic use of ICIs imposes another challenge – the development of immune-related adverse effects. The use of low/ultralow doses of ICI and the development of local delivery systems to avoid systemic distribution of ICIs in breast cancer have shown promise in both pre-clinical and clinical studies. Therefore, future research should focus on the strategy to combine cryoablation with either minimizing the dose or developing local delivery systems to achieve complete elimination of tumor while avoiding adverse effects from prolonged systemic therapies. Combination of cryoablation with immunotherapy may enhance the effect of both therapies for better tumor destruction and long-term tumor-specific immunity against high-risk breast cancers – basically serving as an “*in vivo* cancer vaccine”. Although preclinical mouse and human studies demonstrate a synergistic effect between cryoablation plus immunotherapy, prospective clinical trials are needed to prove this clinical benefit for patients.

## Author contributions

FSM: Conceptualization, Investigation, Writing – original draft, Writing – review & editing. MC: Investigation, Writing – original draft. NR: Investigation, Writing – original draft. SS: Writing – review & editing. RL: Conceptualization, Resources, Writing – review & editing. MM: Conceptualization, Investigation, Supervision, Writing – original draft, Writing – review & editing.
